# Supplementary material for: pH-Dependent Silica Nanoshell Degradation Influences SERRS Enhancement in Biological Environments
Source: ACS Meas Sci Au. 2026 Jul 17;6(4):1141–54. doi: 10.1021/acsmeasuresciau.6c00071 (PMC13495718; doi:10.1021/acsmeasuresciau.6c00071)
Supplement: Supplementary file 1 [file tg6c00071_si_001.pdf]

pH-Dependent Silica Nanoshell Degradation Influences SERRS Enhancement in Biological Environments

William H. Skinner[1], Samuel S. Park,[1] Fay Nicolson*[1]

[1] Department of Radiation Oncology, Dana-Farber Cancer Institute and Harvard Medical School, Boston, MA, 02215, USA

Corresponding Author:

Fay Nicolson

Fay_Nicolson@dfci.harvard.edu

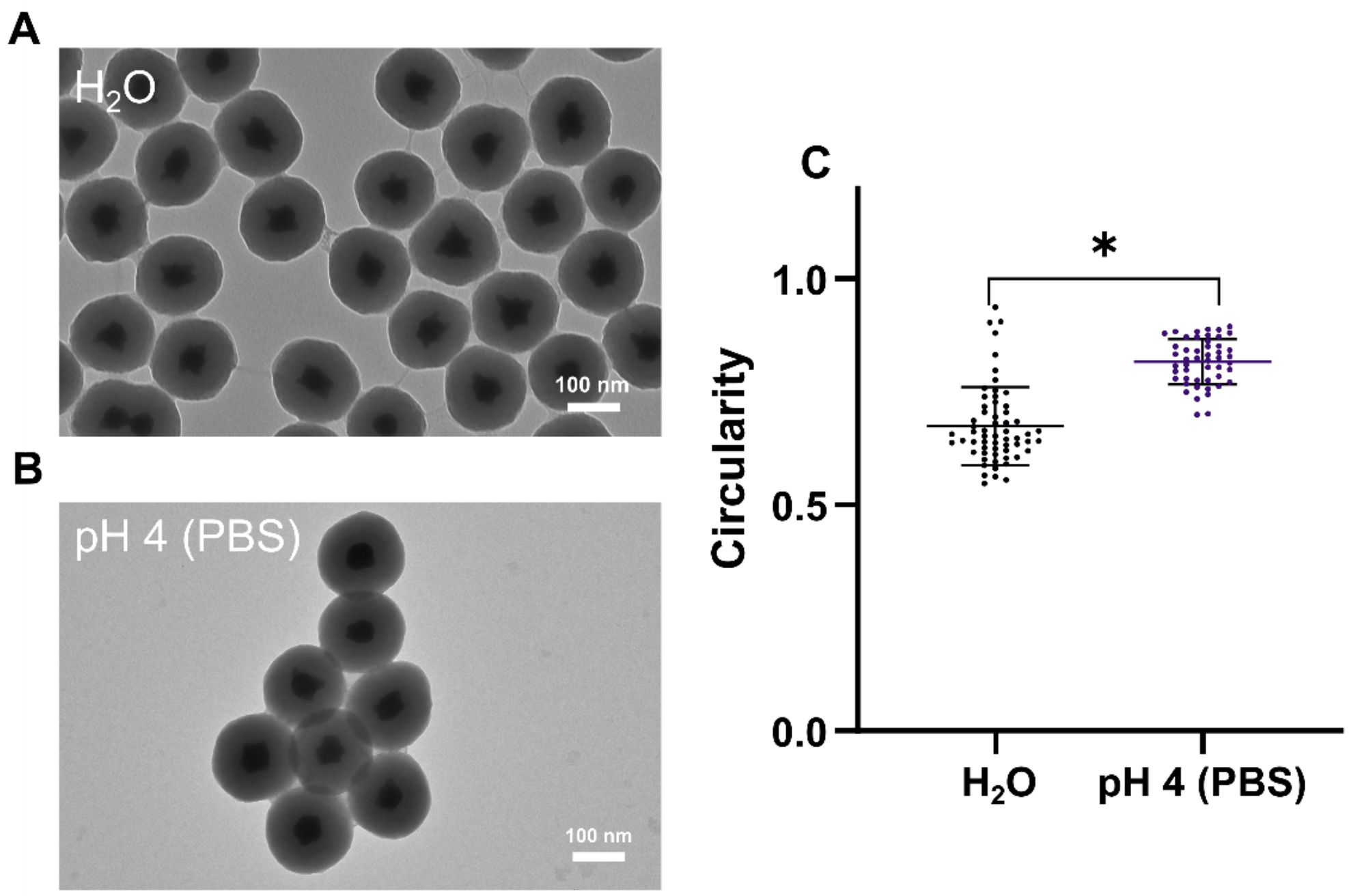

Figure S1: reshaping of silicate gold nanostars within silica shell at pH 4. (A) TEM image of AuNStar-SiO_2 stored in water for 24 hours. (B) TEM image of AuNSTar-SiO_2 stored in PBS at pH 4 for 24 hours. (C) Circularity of AuNStars stored in water and pH 4 PBS for 24 hours. Circularity was measured in ImageJ by highlighting the AuNStar core and using the equation $C = \frac{4\pi A}{p^2}$, where *C* is circularity, *A* is the area of the AuNStar and *P* is the perimeter of the AuNStar.

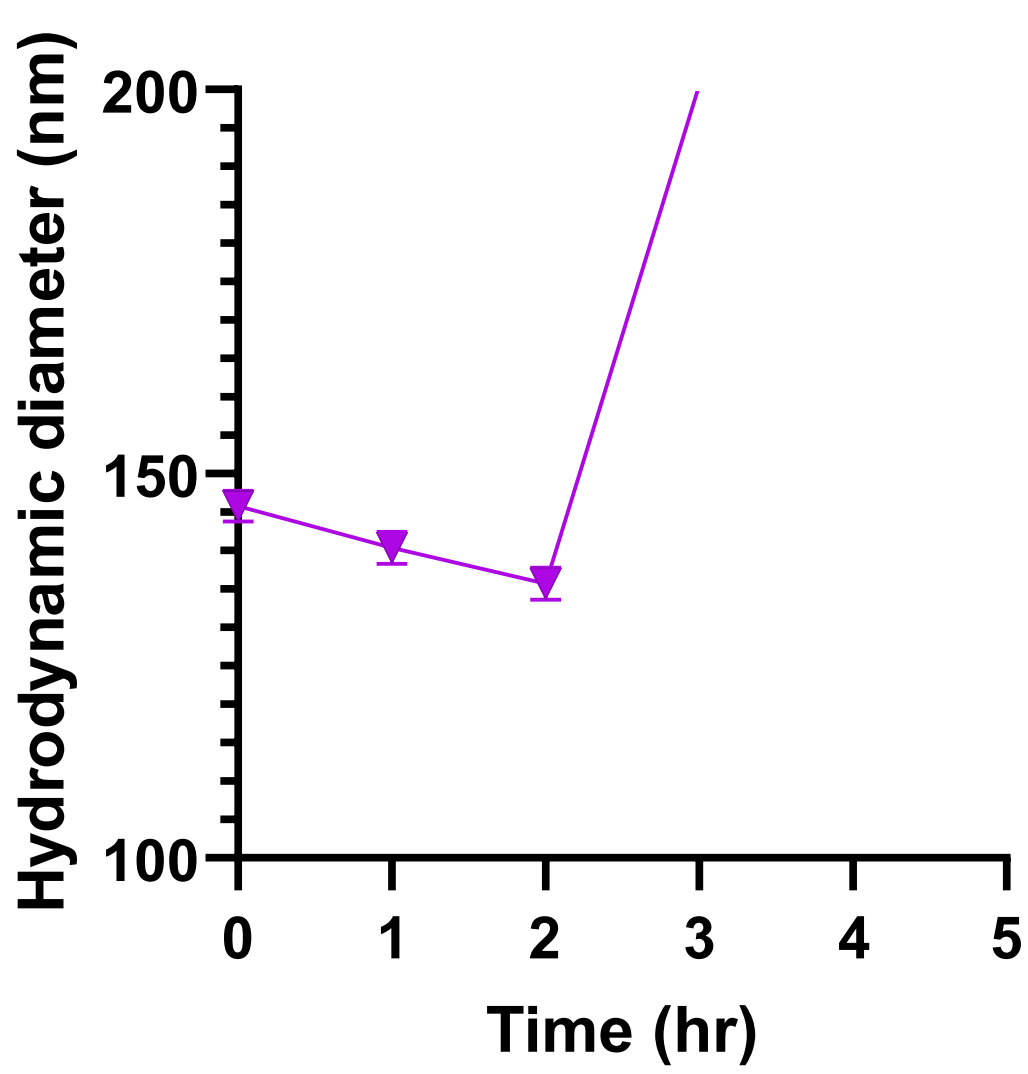

Figure S2: DLS measurements of AuNStar-SiO2 during the first 2 hours of incubation in PBS at pH 7.4. The hydrodynamic diameter initially decreases as the silica shell is hydrolyzed. Nanoparticle size subsequently increases as the released AuNStars aggregate.

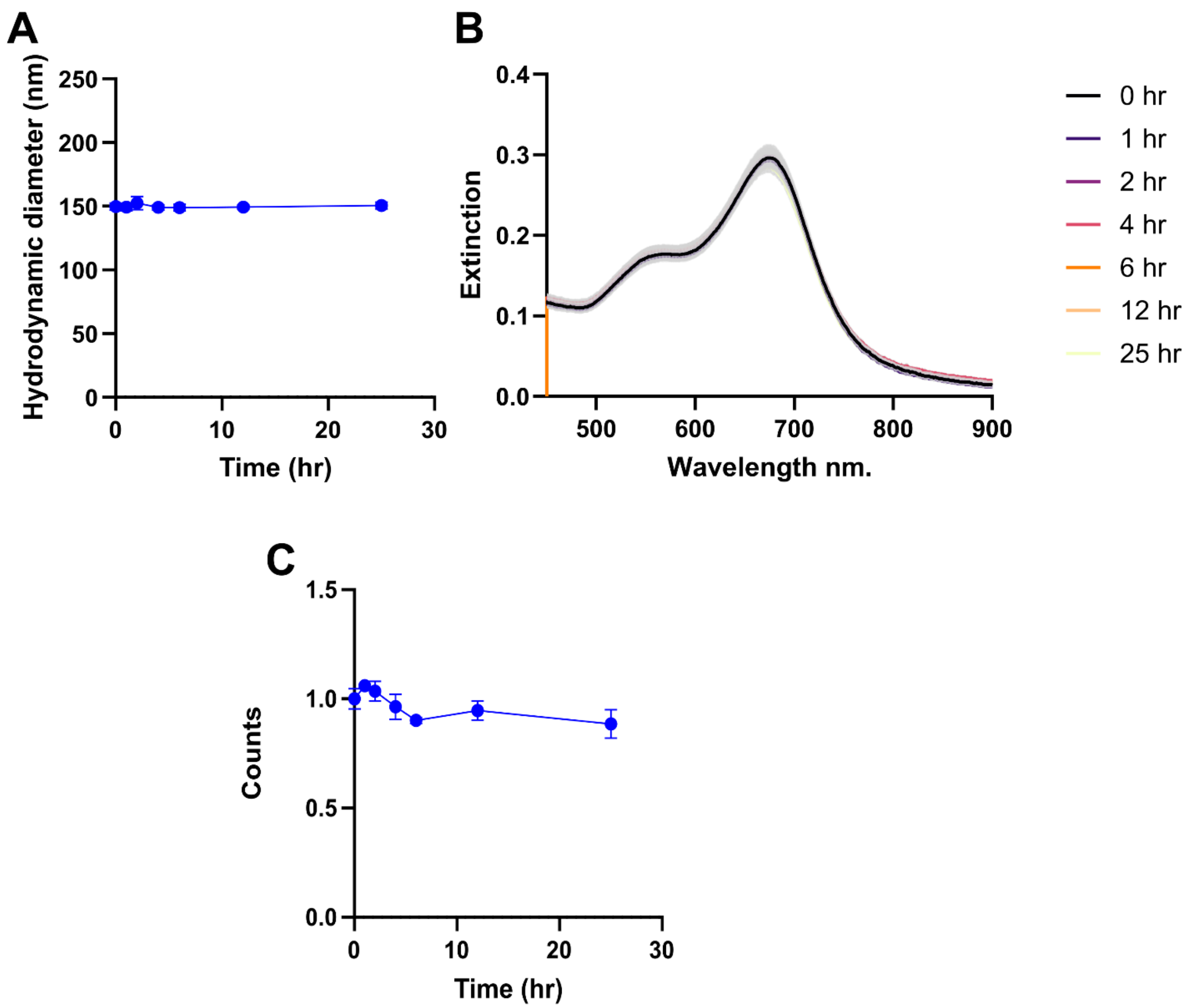

Figure S3: Stability of AuNStars in ultrapure water over 25 hrs, (A) hydrodynamic diameter measured by DLS, (B) UV-vis spectra and (C) SERRS spectra. Three technical repeats were performed per time point.

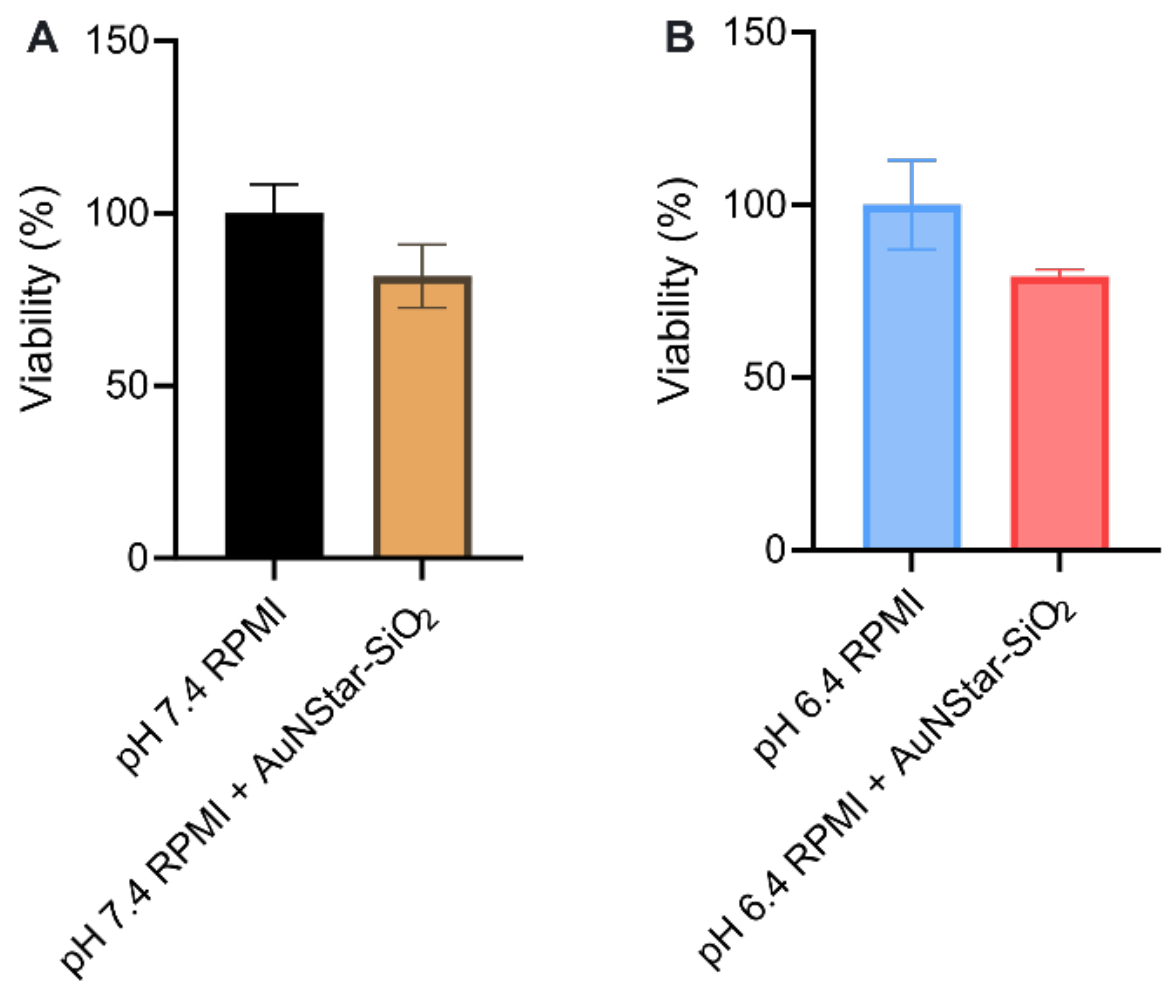

Figure S4: (A) SW48 cell viability after 4 h of treatment with AuNStar-SiO_2 in 0% FBS at pH 7.4. (B) SW48 cell viability after 4 h of treatment with AuNStar-SiO_2 in 0% FBS at pH 6.4.

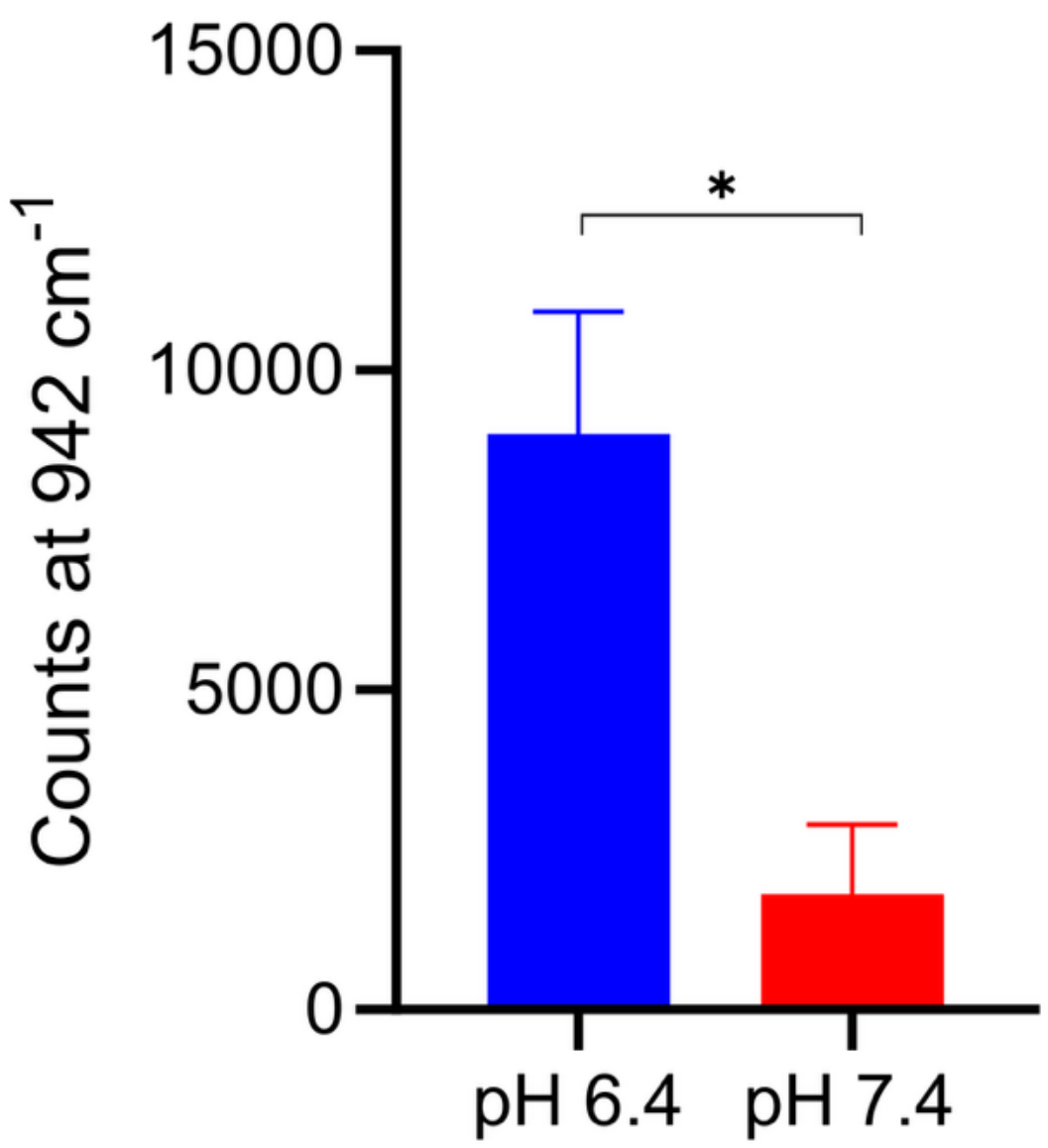

Figure S5: Intensity of SERRS peak at 942 cm-1 from SW48 cells incubated with AuNStarSiO_2 for 4 hours at pH 6.4 and pH 7.4 ($p = 0.02$, n=3).

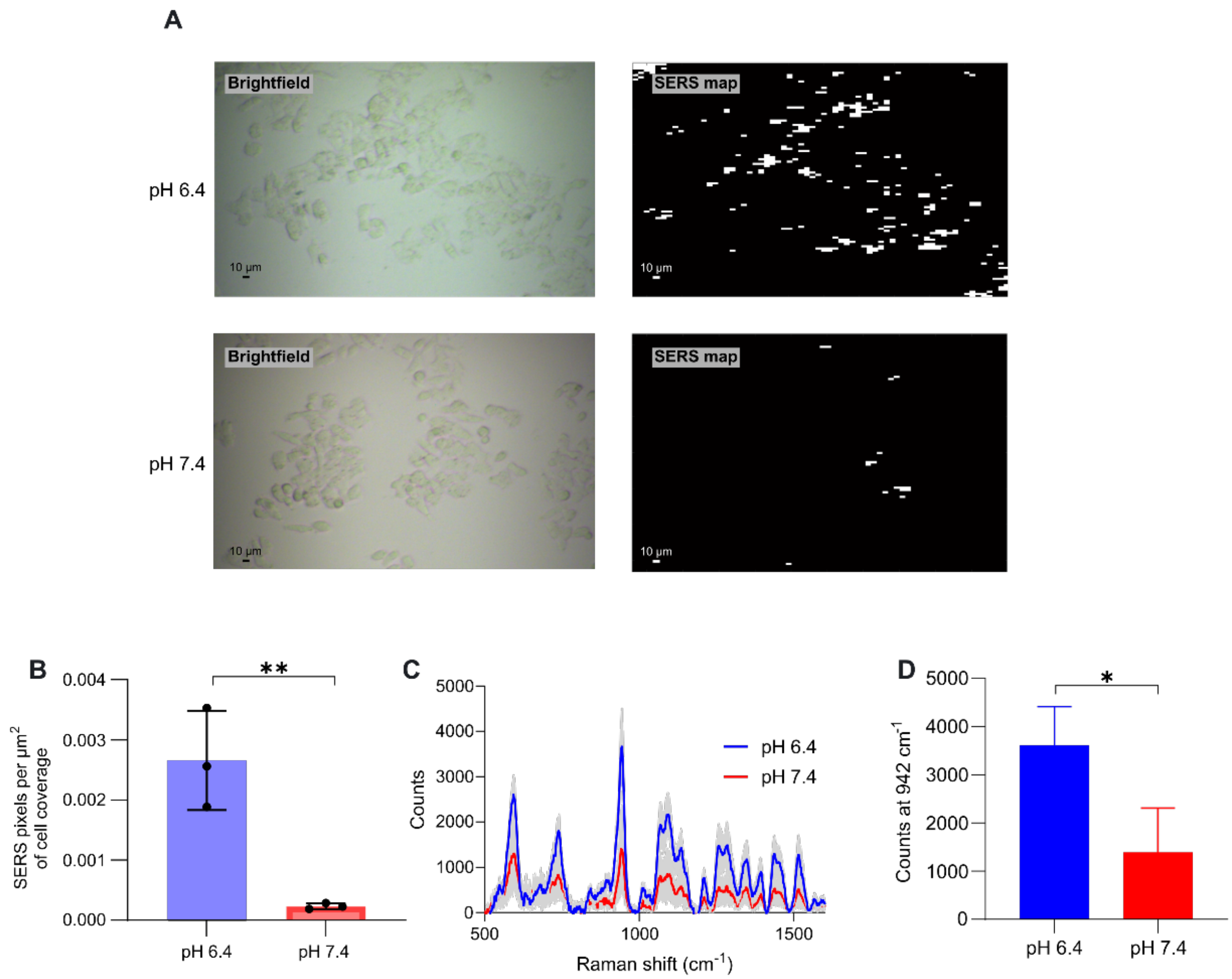

Figure S6: (A) Brightfield and Raman maps of fixed SW48 cells after incubation with AuNStar-SiO_2 at pH 6.4 and pH 7.4 RPMI (phenol-free, 10% FBS). Raman maps were created by masking the cell area in the brightfield and assigning pixels a binary value of 1 or 0 based on whether the spectral intensity at 940 cm^{-1} exceeded 3× the noise level. Maps were collected with a 10x objective, 10% laser power, 0.1 s acquisition. (B) The density of pixels with SERRS intensity > 3× the noise level per µm^2 of cell coverage for cells incubated at pH 6.4 and pH 7.4. Data points are from independent biological replicates. Each data point is the mean of 3 maps from a single sample (t-test, $p = 0.0072$). (C) SERRS spectra of pelleted SW48 cells following incubation at each pH. The shaded area represents the standard deviation of n=3 biological repeats. Spectra were collected with a Raman probe, 785 nm laser, 1 ms exposure time, single acquisition, and a laser power of 40 mW. (D) Intensity of SERRS peak at 942 cm^{-1} from cells incubated with AuNStarSiO_2 for 4 hours at pH 6.4 and pH 7.4 (t-test, $p = 0.03$, n=3).

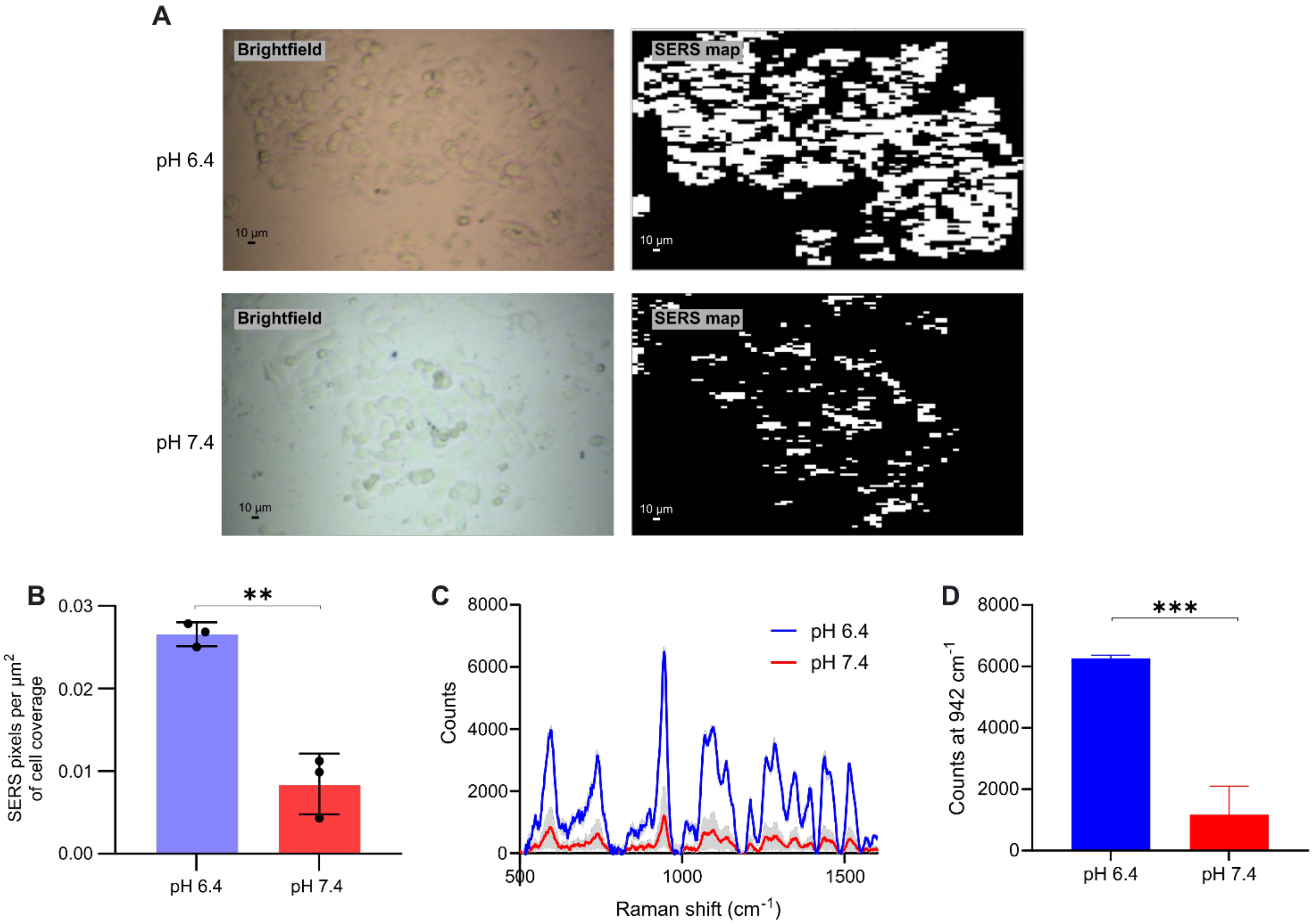

Figure S7: (A) Brightfield and Raman maps of fixed MDA-MB-468 cells after incubation with AuNStar-SiO_2 at pH 6.4 and pH 7.4 RPMI (phenol-free, 0% FBS). Raman maps were created by masking the cell area in the brightfield and assigning pixels a binary value of 1 or 0 based on whether the spectral intensity at 940 cm^{-1} exceeded 3× the noise level. Maps were collected with a 10x objective, 10% laser power, 0.1 s acquisition. (B) The density of pixels with SERRS intensity > 3× the noise level per µm^2 of cell coverage for cells incubated at pH 6.4 and pH 7.4. Data points are from independent biological replicates. Each data point is the mean of 3 maps from a single sample (t-test, $p = 0.0014$). (C) SERRS spectra of pelleted SW48 cells following incubation at each pH. The shaded area represents the standard deviation of n=3 biological repeats. Spectra were collected with a Raman probe, 785 nm laser, 1 ms exposure time, single acquisition, and a laser power of 40 mW. (D) Intensity of SERRS peak at 942 cm^{-1} from cells incubated with AuNStarSiO_2 for 4 hours at pH 6.4 and pH 7.4 (t-test, $p = 0.0007$, n=3).
